# Cellular Senescence - its role in cancer and the response to ionizing radiation

**DOI:** 10.1186/2041-9414-2-7

**Published:** 2011-08-11

**Authors:** Rebecca J Sabin, Rhona M Anderson

**Affiliations:** 1 Centre for Cell and Chromosome Biology and Centre for Infection, Immunity and Disease Mechanisms, Division of Biosciences, Brunel University, West London. UB8 3PH, UK

**Keywords:** Ionising radiation, premature senescence, SASP, inflammation, age-related pathologies

## Abstract

Cellular senescence is a normal biological process that is initiated in response to a range of intrinsic and extrinsic factors that functions to remove irreparable damage and therefore potentially harmful cells, from the proliferative pool. Senescence can therefore be thought of in beneficial terms as a tumour suppressor. In contrast to this, there is a growing body of evidence suggesting that senescence is also associated with the disruption of the tissue microenvironment and development of a pro-oncogenic environment, principally via the secretion of senescence-associated pro-inflammatory factors. The fraction of cells in a senescent state is known to increase with cellular age and from exposure to various stressors including ionising radiation therefore, the implications of the detrimental effects of the senescent phenotype are important to understand within the context of the increasing human exposure to ionising radiation. This review will discuss what is currently understood about senescence, highlighting possible associations between senescence and cancer and, how exposure to ionising radiation may modify this.

## Cellular Senescence

Cellular senescence is a metabolically active form of irreversible growth arrest that halts the proliferation of ageing and/or damaged cells and as a consequence, prevents the transmission of damage to daughter cells. This complicated cellular event is initiated in response to a variety of intrinsic and extrinsic genotoxic stimuli [1-4] and mediated through tumour suppressor pathways involving p53, and p16INK4A/pRb [5,6]. This ultimately leads to the inhibition of cyclin-dependant kinases [7,8]. Accordingly, cellular senescence can be thought of as a tumour suppressor mechanism. Indeed the majority of cancers have mutations in p53 and/or the pRb/p16 pathways, while germ-line mutations in these pathways result in a cell-specific ability to overcome senescence-inducing signals, thereby greatly increasing their susceptibility to cellular transformation [9-11]. The importance of cellular senescence as a tumour suppressor is further demonstrated by cell fusion experiments [12] that provide evidence that growth arrest observed in senescent cells has a strong influence over the growth in proliferating cells and cellular oncogenes of tumour cells. When proliferating cells were fused with senescent cells, DNA replication was inhibited even in the presence of mitogens, and when senescent cells were fused with tumour cells, DNA replication was similarly inhibited. These fusion experiments led to the assumption that senescent cells contained control elements capable of exerting a dominant effect over proliferating pre-senescent cells. Importantly, this tumour suppressive mechanism of cellular senescence has been supported in both mice and human studies [13].

As well as possessing tumour suppressive mechanisms, senescence has been found to play an important role in wound healing and tissue repair and/or communication to surrounding tissues/cells of damage crisis to assist healing [14,15]. For instance, senescent cells have been shown in *in vivo *mouse models to play a role in the resolution of fibrosis by matrix metalloproteinases (MMPs) after acute liver injury. Under normal conditions, proliferating hepatic stellate cells triggered in response to acute liver injury, produce fibrotic scars in advance of entering into a senescent state, followed by secretion of MMPs and scar dissolution. However in cells deficient in either p53/pRb pathways, liver injury results in severe, irresolvable fibrosis [16]. Similarly, the matricellular protein CCN1, which is expressed at sites of cutaneous wound repair, has been shown to initiate DNA damage response pathways and reactive-oxygen species dependent activation of p16INK4A/pRb pathway, resulting in senescence and the expression of anti-fibrotic genes in wild-type mice. Mutant mice that express a senescence-defective CCN1 protein however show increased fibrosis at sites of wound repair [17]. Cellular senescence has been also shown to be important in the prevention of epithelial-mesenchymal transition (EMT) whereby the metastatic dissemination of cancerous cells is prevented [18,19]. Thus, the functional significance of cellular senescence includes a diverse range of roles which are essentially beneficial to the organism.

### Phenotype

The major phenotype of senescence that characteristically distinguishes senescence from quiescence is irreversible growth arrest that cannot be reversed by any known physiological stimuli, associated with resistance to apoptosis and increased sensitivity to cellular injury [20-26]. Other characteristic changes include altered gene expression with increased expression of proteins including p53, p16, p19 and p21 [27,28], an increase in senescence-associated beta galactosidase (SA-β-gal) activity at pH 6.0 [29], the presence of persistent telomere and non-telomere DNA damage foci [30,31], senescence-associated heterochromatic foci (SAHF) [6] and a senescence-associated secretory phenotype (SASP) [32,33]. Accordingly, identification of senescent cells can be achieved by assaying for a combination of the above characteristics. The application of proliferation cell-cycle specific markers, such as Ki-67, can also be used [34-36]. For example Kill *et al *(1996) showed that 56% of human dermal fibroblasts were Ki-67 positive at early passage 4 [37] compared to only 30% at passage 38 [38] with the decrease in fraction of Ki-67 positive cells reflecting an increase in senescence.

A range of morphological changes have also been documented with senescent fibroblasts showing enlarged and flattened morphology accompanied by the loss of elongated, spindle-like properties, when compared to normal proliferating fibroblasts. Specifically, the mean nuclear area of fibroblasts was shown to be 255 μm^2 ^at early passage, compared to 293 μm^2 ^at later passage [39]. Interestingly, the sub-nuclear organisation of chromosomes has also been shown to be different in senescent and proliferating mammalian somatic cells, whereby gene poor chromosomes such as chromosomes 13 and 18 are thought to alter their preferential nuclear position from near the nuclear periphery and relocate to the nuclear interior when induced to senesce. Thus, the interphase organisation of particular chromosome territories changes such that their position correlates according to the size of the chromosome, rather than the density [40,41].

### Replicative Senescence

Seminal work carried out by Hayflick and Moorhead (1961) demonstrated that normal cells grown in culture dishes are only able to undergo a finite number of cellular divisions before their growth is irreversibly arrested [42-44]. This 'Hayflick Limit' was the first demonstration of a senescence phenotype and described the replicative capacity of diploid cells in culture before the cells ceased to divide [45] and has since been demonstrated for many different types of cells both *in vitro *and *in vivo *[46,47]. Importantly, diploid cells within cell populations do not all reach senescence at the same time, rather there is a progressive decrease in the fraction of proliferating cells that are capable of undergoing cellular division with each round of replication [48,49]. Also, different cell types and lineages will vary in the rate at which they enter a senescent state [50]. For example, *in vitro *studies comparing the growth rates and passage number of fibroblast and keratinocyte cell types observed that the decline in cell growth rate was notably higher for keratinocytes which had senesced by P6, compared to fibroblasts that were passaged beyond P10 in all donor age groups above and below 40 years of age; suggesting that the growth rate of the two cell types is independent of donor age [51]. Indeed a 'memory' for the number of completed population doublings was observed when WI-38 fibroblasts were found to enter senescence with respect to their remaining replicative capacity, even after cryopreservation for a period of 23 years [44].

The mechanism for replicative senescence is believed to be associated with a progressive shortening of telomeres that occurs with each DNA replication cycle [52,53]. Functional telomeres protect the ends of chromosomes however, approximately 100-300 bp of these repeat sequences are lost as a result of incomplete replication of the extending 3' overhang of nucleotides [54,55]. Support for this comes from studies that show critically short telomeres trigger a DNA damage response which results in cellular senescence [30,56], while immortal cancer and germ cell lines overcome the action of telomeric shortening by the expression of the telomerase enzyme [57]. Telomerase synthesizes and maintains telomeric end sequences, preventing the exposure of uncapped ends [58] thereby permitting continued cellular proliferation [59-63].

### Stress-induced Premature Senescence (SIPS)

Stress-induced premature senescence (SIPS), also known as premature senescence, culture shock and STASIS (stress or aberrant signalling-induced senescence) [64-66], occurs rapidly in response to a variety of intrinsic or extrinsic stressors, including DNA damage from ionising and non-ionising radiation, cytotoxic DNA damaging agents, oxidative stress and as a consequence of oncogenic activation [3,4,8,67]. Serrano *et al *(1997) were among the first to identify a form of SIPS that was not attributable to telomere attrition that they described as oncogene-induced senescence (OIS). The group showed that oncogenic ras expression permanently arrested primary human and rodent cells in G_1 _and that the cells displayed features similar to those of replicative senescence, including the accumulation of p53 and p16 [65]. Importantly the expression of the catalytic subunit of the telomerase enzyme hTERT has been shown not to abrogate SIPS, demonstrating that cellular senescence can be triggered prematurely independently of telomere attrition [68,69]. Thus, oncogenic activation and stressors that lead to DNA damage but which are independent of loss or dysfunction of telomeres, can initiate a response that results in a cellular phenotype indistinct to that observed for replicative senescence [63,70].

### Molecular Pathways of Senescence

**Figure 1:**
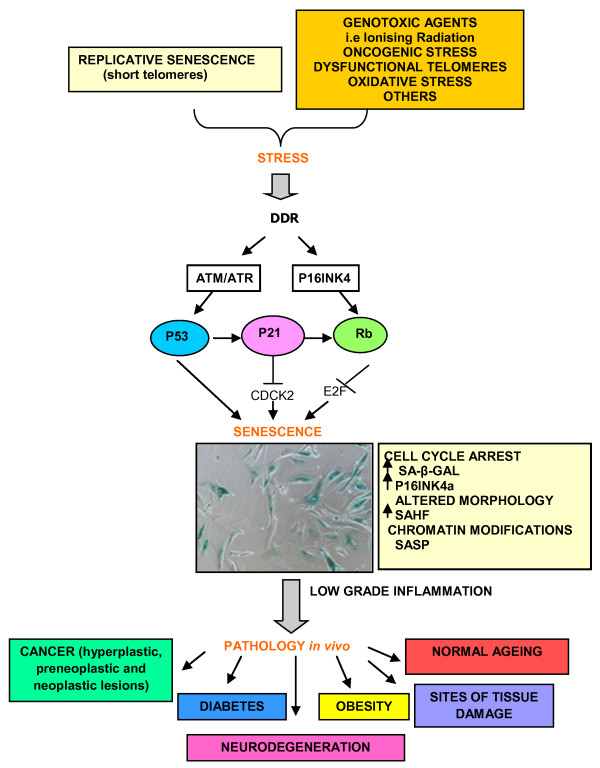
**Scheme highlighting initiating and molecular mediators of cellular senescence**. The senescent phenotype includes expression of SA-β-galactosidase (SA-β-gal), increased expression of p16INK4a leading to cell cycle arrest and an increase in the secretion of pro-inflammatory factors termed as senescence-associated secretory phenotype (SASP). Senescent cells have been observed in normal ageing cells and in cells/tissues of various age-related pathologies.

The initiating event for both replicative senescence and SIPS involves the recognition of DNA damage and the activation of the DNA damage response (DDR) pathway [3,56,71-74]. The key mediator in this process, ATM, phosphorylates important sensors and effectors of the DDR including H2AX, 53BP1 [31,75-77] and p53 leading to the up-regulation of cyclin-dependant kinase inhibitor p21, which in-turn acts to inhibit the action of CDK2 kinase activity arresting the cell cycle in G_1 _[76] (Figure 1). In addition, p21 also activates pRb through the inhibition of cyclin E/CDK2 [1]. Where SIPS differs from replicative senescence is in the formers dependence on the P16INK4 family of tumour suppressor proteins, which are activated upstream to pRb [10,78]. Accordingly, increased P16INK4A expression is considered as another useful marker of senescence *in vitro*, and indeed elevated protein levels have been detected in ageing baboon fibroblasts along with markers of telomere damage and SAHF [79]. The hypophosphorylated state of pRb results in inhibition of the transcription factor gene E2F and this acts to bring about G_1 _cell cycle arrest. For this reason, the p53 and p16/pRB dependent senescent pathways are not completely separable and as well as the common link through p21, pRB has been shown to regulate the activity of MDM2 which acts to control the stability of p53 [80]. Thus increased expression of p21 is important for senescence [81]. Consequently, the DNA damage response, apoptotic and senescence pathways share common molecular mediators through p53 and p21. What directs a cell to senesce or apoptose remains unclear, but cell type, the type of damaging agent and the dose administered may be important; as well as the post-translational modifications that p53 undergoes [82]. For instance in normal cells, senescence has been shown to be more favourable than apoptosis to deal with low levels of DNA damage, perhaps as the cell makes the decision to attempt to repair instead of removal from the cell pool [3,76,83]. By contrast, adult human dental pulp stem cells (DPSCs) were found to enter premature senescence in the G_2 _phase of the cell cycle after exposure to much higher doses (2-20 Gy) of ionising radiation, as detectable by phosphorylated p53 and increasing p16 expression observed over 13 days and SA-β-gal activity from day 3 after irradiation [84]. Possible mechanisms that may be involved in determining cellular fate include the status of the tumour suppressor phosphate and tensin homolog (PTEN). For instance, Lee J, *et al *(2010) showed that PTEN-deficient glioma cells preferentially entered senescence, while PTEN-proficient glioma cells generally apoptosed in response to ionising radiation. The authors concluded that SIPS may be a compensatory mechanism in place of apoptosis when PTEN tumour suppressor protein is absent [85].

What is clear is that the convergence of multiple pathways through p53 and pRb are required to establish and maintain the senescent state and removal of either of these has been shown to prevent senescence in mouse embryonic fibroblasts [86]. In humans, it is thought that both p53 and pRb pathways must be inactivated in order to prevent the onset of cellular senescence [87], consistent with the majority if findings that show ~50% of all tumours show evidence of mutated/non-functional p53 and/or pRb [88,89]. Interestingly though, more recent experiments in humans and mice have shown that senescence can be prevented, or significantly delayed as a result of inactivation of either p53 or Rb alone while some cell types exhibit delayed onset of senescence upon p16 inactivation [90]. Therefore the relationship between these two pathways and the potential for redundancy in either pathway may provide further protection against senescence bypass in different cell types [3,91].

## Senescence and Cancer

Senescence, in addition and in contrast to the previously noted beneficial tumour suppression and tissue repair effects, has also been linked to reduced tissue functionality and is increasingly thought to play a role in age-related pathologies such as cancer, Alzheimer's disease, diabetes and obesity [54,92-96]. For instance, senescent cells have been observed in many ageing mice tissues [20,97], baboon skin fibroblasts [79] and human tissues [29] indicating that senescence may have a causal role in ageing *in vivo *as well as *in vitro*. Senescent endothelial cells have been shown to increase in atherosclerosis, thrombosis and at sites of inflamed vascular endothelium [18,98], demonstrating possible links with pathology. Therefore, the accumulation of senescent haemopoietic stem cells has been suggested as a possible mediator for the decline in tissue regeneration and repair with age.

Markers of DNA damage are known to accumulate in ageing stem cells [64,99-101] and other senescing human and mouse cell types [102-104]. For instance, studies employing the DSB marker, γ-H2AX, reveal γ-H2AX foci to accumulate in normal human fibroblasts, WI38 fibroblasts and PrEC prostate epithelial cells with increasing passage in a manner that correlates with an increasing fraction of SA-β gal positive cells. Specifically, early passage cultures show 0.2-0.3 γ-H2AX foci/cell increasing to 2.2-4.1 foci/cell in senescent cultures [31]. Further to this, radiation-induced γ-H2AX foci have both been shown to increase in both murine and human senescent cells *in vitro *[56,73,74] while, *in vivo *studies have shown the long-term expression of senescence markers, including an increased expression of p16INK4a to be coupled with the persistence of DNA damage foci 45 weeks post irradiation to a sub-lethal dose of radiation [105]. Interestingly, γ-H2AX foci (along with other DNA damage markers such as 53BP1) have been shown to localise both at telomeres as telomere-dysfunction-induced-foci (TIFs) in both early and late passage fibroblasts [106] and also throughout the genome as a consequence of ionising radiation exposure. Therefore γ-H2AX foci seen in senescent cells are not necessarily telomere-associated foci, representative of replicative senescence, but may represent SIPS-induced sites of DSB, highlighting that mediators of SIPS may contribute to age-related pathologies, including cancer. Accordingly, there is a relationship between cellular ageing and the accumulation of residual DNA damage both *in vitro *and *in vivo*, however as yet there is no evidence to determine whether senescence is a resultant part of ageing and age-related pathologies or whether it is a state that contributes to the development of ageing tissues and tissue pathology. Interestingly though, mouse models of accelerated ageing that are deficient for p16INK4a show delayed onset of age-related phenotypes, highlighting the role of increasing p16INK4a in maintaining the senescent state and its role in age-related decline of tissue regeneration and repair [107]. Further studies will hopefully decipher the evidential link between increasing populations of senescent cells and the contribution they have in the development of age-related pathologies such as cancer [108,109].

### Senescence-Associated Secretory Phenotype (SASP)

It is well established that senescent cells secrete factors such as interleukins, chemokines, growth factors and proteases, encompassing what is known as the senescence-associated secretory phenotype (SASP) [15,32,110]. The function of SASP is to mediate the characteristic growth arrest of senescence via the autocrine activities of pro-inflammatory cytokines (including IL-6 and IL-8), in addition to pro-apoptotic protein insulin growth-factor binding protein 7 (IGFBP7), epithelial growth factors (heregulin and VEGF), matrix metalloproteinases including MMP-3 and plasminogen activator inhibitor 1 (PAI-1) [111-113]. Interestingly the name 'senescence-messaging secretome' (SMS) was proposed to highlight that the associated factors of the secretory phenotype were not only essential for initiating the senescent state but also for its maintenance and communication of this state to the local microenvironment [114,115]. A study that highlights this communication shows that re-activation of endogenous p53 in p53-deficient tumours in a mosaic mouse model of hepatocellular carcinoma led to tumour regression. This was proposed to occur through the induction of cellular senescence and up-regulation of inflammatory cytokines, triggering an innate immune response *in vivo *that ultimately led to tumour clearance [116]. Thus, inflammatory cytokines are necessary for both the establishment and maintenance of senescence, suggesting SASP/SMS products are important for the suppression of malignancy [111,114]. However, SASP is also known to influence the proliferation of neighbouring cells and disrupt tissue architecture [117], principally through these pro-inflammatory influences. For instance, an increase in VEGF, as a result of senescent fibroblasts, has been seen to stimulate tumour vascularisation and invasion of basement membranes [118]. Further, inflammation is thought of as a key mediator in cancer development and inflammatory cytokines and MMPs are being increasingly implicated as a contributing factor in this multistep process.

What this suggests is that senescent cells can actually promote, in addition to preventing, the progression of malignancy; a relationship that is described as antagonistically pleiotrophic [21,118-121]. For instance, senescent human fibroblasts have been shown to stimulate pre-malignant and malignant fibroblasts to hyperproliferate and form tumours in mouse models when senescent cells comprised ~10% of the fibroblast population [122]. Close proximity of senescent fibroblasts to pre-neoplastic cells are thought to be the trigger for this change. Additionally, after exposure to the DNA-damaging agent bleomycin, human SIPS fibroblasts co-transplanted into xenografts of immunodeficient mice were seen to stimulate nearby cancer cells to proliferate, either directly or through local tissue damage and inflammation mediated by MMPs [123]. These findings support SASP as being an important mediator of the transformation process of pre-neoplatic cells. In addition, studies carried out by Zhou *et al *(2011) have shown SIPS in normal airway epithelial cells to result in an impairment of repair of drug-induced damage initiating a p38 MAPK dependant increase of pro-inflammatory cytokines that was subsequently seen to exacerbate the airway injury [124]. Interestingly this cytokine secretion, which primarily involves IL-6 and IL-8, is only established as a result of persistent DNA damage response signalling (DDR) and not as a result of transient signalling [125] suggesting the presence of long-lived, irreparable DNA lesions are important in this process.

Thus alteration of the tissue microenvironment that results in the promotion of cell growth as a consequence of the senescence phenotype, through inflammation and persistent tissue damage [4,15,126] may provide a mechanism whereby senescent cells may also contribute to cancer promoting effects in otherwise normal tissues [127]. If demonstrated then senescence may functionally protect young animals from cancer via tumour suppression, whilst contributing to the deleterious effects in aged organisms through persistent inflammation and tissue injury [122,126].

## Ionising radiation and Senescence

Ionising radiation is known to induce SIPS in both normal and cancer cell types after exposure to relatively high doses (10Gy) of radiation [125,128-131]. Thus, an important implication is what contribution, if any, senescence plays as a possible mediator of tumour recurrence after radiotherapy, given the effects of SASP in stimulating pre-neoplastic cells as discussed earlier. SIPS is also induced after exposure to lower doses of radiation [125,130,132,133] which similarly has consequences for understanding human cancer risk to radiation exposure, but this time within the context of SIPS in normal tissue after e.g. diagnostic exposures. For instance it is well established that radiation induces damage in cells that are not directly irradiated but which are in communication with irradiated cells. This radiation-induced non-targeted bystander (NTE) phenomenon is known to dominate at low radiation doses and to mediate a range of cellular effects such as DNA damage [134,135], cell death [136], cell proliferation, adaptive protective effects and malignant transformation [75,137-141]. To date, such NTE have been observed in microbeam-irradiated human tissue [141,142], *in vivo *animal models [143-145] and interestingly, in cells cultured in both non-irradiated tumour and senescent cell conditioned medium [14,75].

Thus, it is reasonable to ask if there is a possible concordance between radiation-induced SIPS and SASP, and candidate mediators of NTE effects. Reactive oxygen species (ROS) are known to be important damaging agents involved in NTE [143,146,147], but additionally, activated macrophage, NO, IL-6, IL-8, IFN-γ and TGF-β have all also been implicated [143,144,148]. For instance, one study used radiation-induced AML susceptible and resistant mouse strains, CBA/ca and C57BL/6 respectively, to correlate radiation-induced up-regulation of gene expression of a M1 pro-inflamatory macrophage profile with more NTE in CBA/ca and an M2 anti-inflammatory macrophage profile, with less NTE in C57BL/6 strains [144]. Thus, candidate sources for the mediation of radiation-induced NTE include inflammation-associated cytokines and chemokines secreted from irradiated (or otherwise stressed) cells. Whether the irradiated cells that contribute to this plethora of inflammatory signals remain within the proliferative pool upon repair of damage or whether they become senescent is unknown however it is clear that even low doses of radiation induce SIPS and these cells subsequently secrete inflammatory cytokines, including IL-6 and IL-8 [27,110,133,149-153]. Interestingly Tsai *et al *(2009) showed that stromal fibroblasts that were induced to senesce after low dose radiation exposure stimulated the proliferation of breast-carcinoma cells when co-cultured in the same medium [125,130,132,133].

This potential relationship between exposure to radiation, cellular age and deleterious inflammatory (NTE) responses is further demonstrated by human and animal studies which show a correlation exists between the immunological imbalances caused as a result of exposure to radiation and, those effects which are seen in normal aged immune cells, implying ionising radiation may accelerate immunological ageing [154]. For instance, the normal age-related decrease of total CD4^+ ^T-cells was found to be ~4% per 10 years, compared to a radiation-induced decrease of ~2% Gy^-1^, equivalent to a 5 year age increase per 1 Gy [155,156]. This group also demonstrated a dose-dependent increase in CD25^+^/CD127^- ^regulatory T-cells and attributed T-cell immunosenescence to a higher level of inflammatory markers in A-bomb survivors. For instance, changes in the immunological profiles of cytokines, known to be involved in the coordination of the inflammatory response (TNF-α, IFN-γ, IL-6 and IL-10) were seen in both A-bomb survivors and liquidators which may contribute to the persistent subclinical inflammatory status that is seen in these individuals [157-160]. There is the suggestion therefore that radiation-induced enhancement of inflammatory reactions might contribute to the development of radiation-induced disorders and premature ageing [155,161]. Indeed, it is also well known that A-bomb survivors show increased cardiovascular and respiratory diseases associated with persistent inflammation [162,163].

Taken all together it is tempting to speculate radiation-induced SIPS and SASP as important mediators and, or amplifiers of radiation-induced NTE, which in turn may perpetuate inflammatory signals that subsequently, also contribute to increasing SIPS. In elucidating the importance of any such relationship in contributing to cancer risk, particularly at low doses, future work needs to understand the relevance of radiation quality, dose and dose rate in initiating SIPS and the long term tissue damage and pathological alterations that may arise as a consequence.

## Conclusion

The beneficial tumour suppressive role of senescence whereby damage is prevented from being transmitted to daughter cells is well established. What is only recently becoming apparent is that pro-inflammatory factors such as those encompassing the senescence-associated secretory phenotype (SASP) are linked to cellular proliferation, a persistent low grade inflammation, elevated DNA damage foci and transformation of pre-neoplastic cells. Human populations are increasingly being exposed to ionising radiation from a range of diagnostic, treatment and occupational sources highlighting the potential risks of SASP whereby stress-induced premature senescence (SIPS) is initiated instead of apoptosis. The potential effects of this are two-fold; accelerated cellular ageing and an amplification of any detrimental effects produced by SASP. Thus, further research is required to understand the relationship between exposures to radiation, SIPS and how, in turn, SIPS may modify the biological effect of radiation exposure.

## Competing interests

The authors declare that they have no competing interests.

## Authors' contributions

RJS drafted the manuscript. RA participated in its design and helped to draft the manuscript. Both authors read and approved the final manuscript.
